# Follicular Adenomatoid Odontogenic Tumor: A Case Report

**DOI:** 10.7759/cureus.64168

**Published:** 2024-07-09

**Authors:** Suwarna Dangore-Khasbage, Swapnil Mohod, Ravikant Sune, Aayushi Pakhale, Aakanksha Tiwari

**Affiliations:** 1 Oral Medicine and Radiology, Sharad Pawar Dental College and Hospital, Datta Meghe Institute of Higher Education and Research, Wardha, IND; 2 Oral Pathology and Microbiology, Sharad Pawar Dental College and Hospital, Datta Meghe Institute of Higher Education and Research, Wardha, IND

**Keywords:** two-third tumor, intraosseous, odontogenic, pericoronal, adenomatoid odontogenic tumor

## Abstract

Adenomatoid odontogenic tumor (AOT) is an infrequent odontogenic tumor that typically occurs in adolescent females, usually in the anterior maxilla. There is a controversy about it being a tumor or a hamartoma. It presents clinically as a slowly progressive entity that shows a good prognosis with conservative surgical management. It shows three clinicopathological types: follicular, extrafollicular, and peripheral. This article describes a follicular variety of AOT. An 18-year-old female presented with diffuse intraoral swelling in the maxillary anterior region. An intraoral periapical radiograph (IOPA) revealed a single large pericoronal radiolucency related to the impacted right maxillary lateral incisor. Histopathological evaluation confirmed the diagnosis, and the tumor was treated surgically by enucleation.

## Introduction

Oral and maxillofacial tumors have a diverse range of clinical characteristics, as well as histopathological presentations, and thus represent a heterogeneous pathologic disease. Among these, benign odontogenic tumors are the slow-growing lesions causing bone expansion, the displacement of the teeth, and damage to surrounding craniofacial structures. They have a great variety in their presentation, either clinically or radiographically. Basically, early detection is vital as orofacial tumors are associated with morbidity and mortality [1]. Benign odontogenic tumors are highly curable entities. First-line surgical therapy must be planned logically and appropriately. Long-term postoperative follow-up is generally recommended for all patients with odontogenic tumors so as to ensure effective tumor control [2].

Adenomatoid odontogenic tumor (AOT) is one of the fairly infrequent benign odontogenic tumors with a prevalence of around 3%-7% and reported by a number of synonyms in the literature [3]. Philipsen and Birn identified it as a different entity and named it AOT in 1969 [4]. It is also called a "two-third tumor," considering its prevalence in the maxilla, association with canine, association with unerupted tooth, and female prevalence in two-thirds of cases [5,6]. These are frequently small and are not known to cause root resorption of adjacent teeth.

Nevertheless, rare cases with large sizes causing the displacement and resorption of the tooth roots, which are in the vicinity, are also reported [7]. This entity usually does not depict typical clinico-radiological appearances. Usually, it is managed by a conservative approach considering a low recurrence rate [8].

## Case presentation

**Figure 1:**
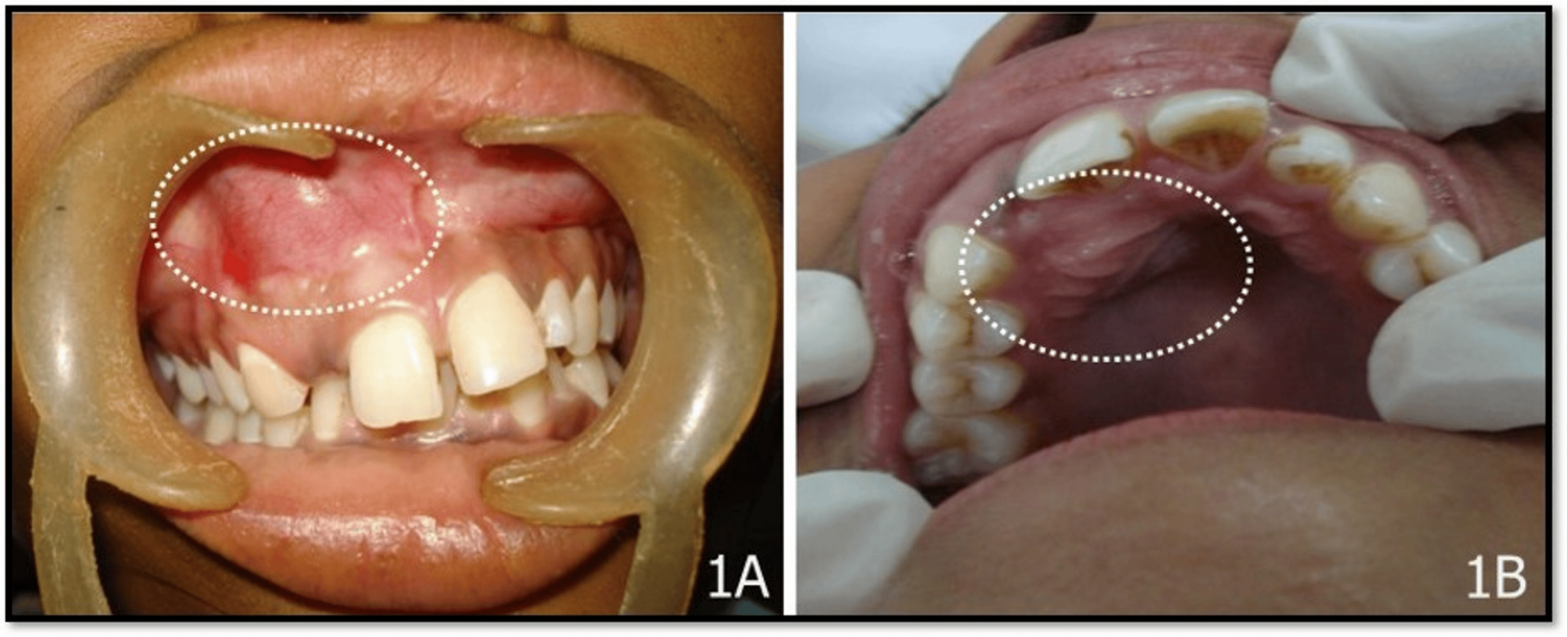
Clinical image depicting (A) swelling labially in the maxillary right central incisor to the canine region and (B) swelling palatally in the same region

An 18-year-old female reported to the dental department for poor aesthetics due to a missing anterior tooth and swelling in the front region of her upper jaw for the past 10 months. Intraoral examination showed swelling on the labial and palatal aspect of the maxillary right central incisor. The borders of both labial and palatal swelling were diffuse, while on the labial aspect, there was an obliteration of the labial vestibule in the same region (canine and clinically missing maxillary right lateral incisor as shown in Figure 1A, 1B).

**Figure 2:**
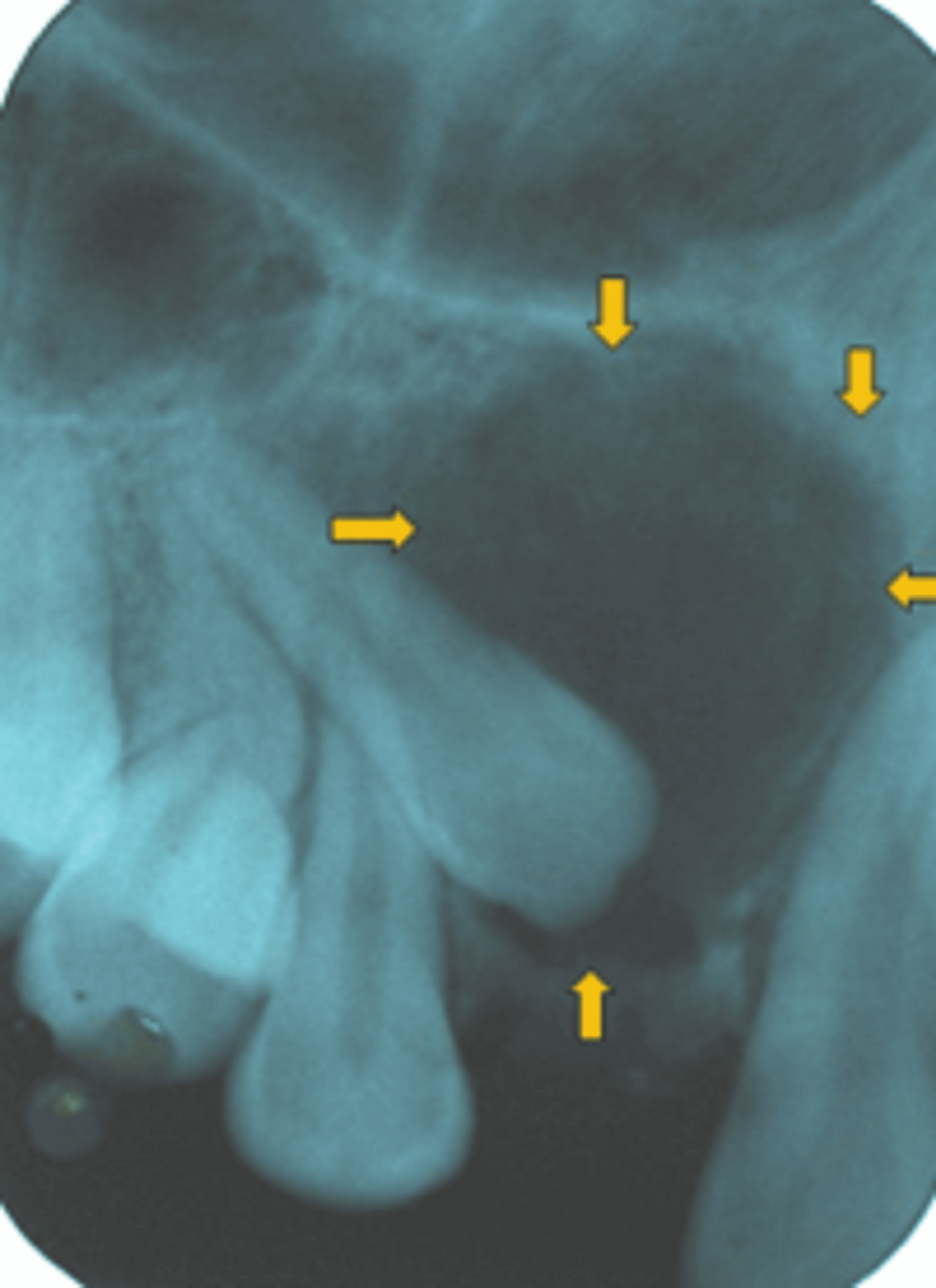
IOPA revealing pericoronal radiolucency associated with unerupted right maxillary lateral incisor IOPA: intraoral periapical radiograph

The covering mucosa appeared normal. On palpation, the swelling was hard in consistency, suggestive of an expansion of the bone due to the pathology. It was not tender, not fluctuant, and without any eggshell crackling. No abnormalities were detected with the teeth in the vicinity of the swelling. These were non-tender, nonmobile, and vital. No other intraoral abnormality was detected except the crowding of the mandibular anterior teeth. With the history of slow-growing lesions and the characteristic location of the lesion in the maxillary anterior region, AOT was considered a provisional diagnosis, while with dentigerous cyst with clinically missing maxillary right lateral incisor, odontome and calcifying odontogenic cyst were well-thought-out in differential diagnosis. As a first step in the investigation, an intraoral periapical radiograph (IOPA) of the same region was taken that revealed the presence of an impacted maxillary right lateral incisor with a well-defined pericoronal radiolucency. The impacted maxillary right lateral incisor was distally displaced, while other teeth in the vicinity revealed no displacement. There was no evidence of calcific foci within the radiolucency. Radiolucency was not attached to the cementoenamel junction; rather, the root of the maxillary right lateral incisor was partially within the radiolucency. Maxillary right canine was not affected; however, there was an overlapping of impacted maxillary right lateral incisor over the canine root (Figure 2).

**Figure 3:**
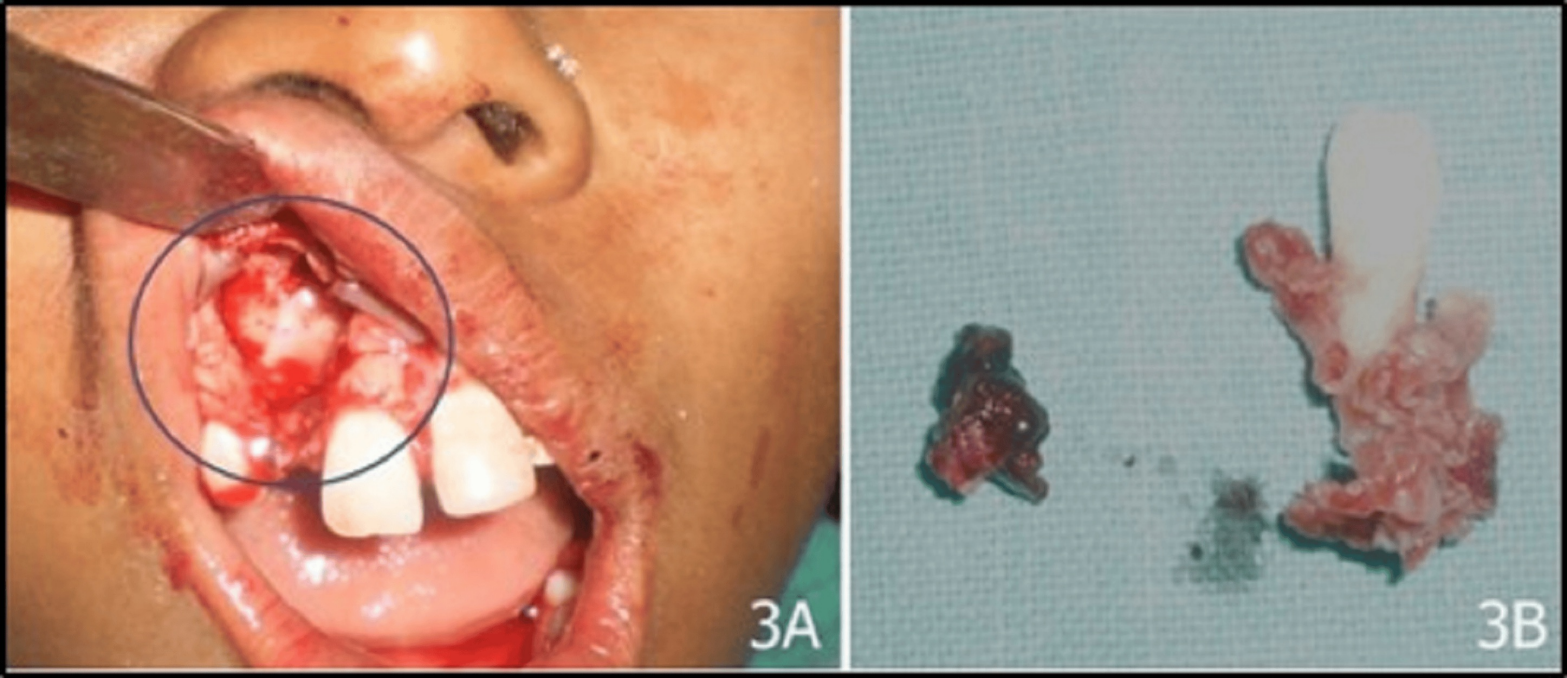
(A) Surgical site after the removal of the lesion and (B) excised specimen and tissue along with attached maxillary right lateral incisor

Considering the clinico-radiographic features such as female gender, being a teenager, the maxillary anterior region, and well-defined pericoronal radiolucency, the diagnosis was reported to be a follicular variety of AOT. Nonetheless, dentigerous cyst and calcifying epithelial odontogenic cyst were the radiographic differential diagnoses. Further aspiration cytology from the swelling was performed that cleared the picture, as it was nonproductive and aided to rule out cysts. The lesion was treated conservatively with enucleation with bone curettage and the extraction of the maxillary right lateral incisor (Figure 3).

As there was malocclusion evident in clinical examination, orthodontic treatment was advised for the correction of malocclusion, and the replacement of the missing tooth was planned by doing a fixed prosthesis.

**Figure 4:**
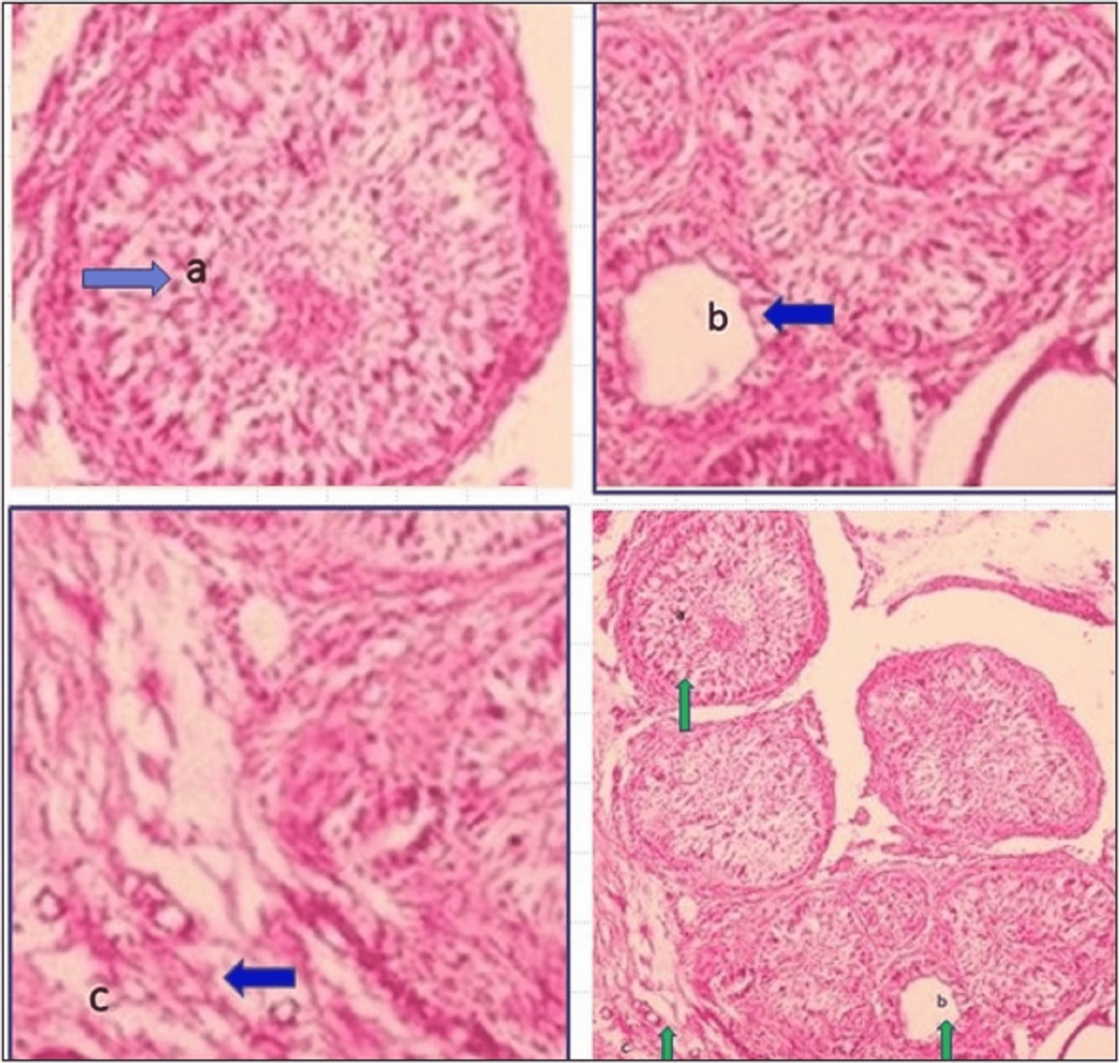
(a) Rosette-like proliferation of odontogenic cells, (b) odontogenic cells forming duct-like structure, and (c) fibrous capsule (d) showing all these features (H&E: 20×)

The excised specimen was examined histopathologically, which revealed the presence of lesional tissue with the proliferation of cuboidal and columnar cells in multinodular pattern forming nest- or rosette-like structures. Duct-like structures were seen lined by cuboidal to columnar odontogenic cells with nuclei polarized away from the lumen. The lesional tissue was surrounded by a fibrous capsule. Features were suggestive of AOT (Figure 4).

The patient is on regular follow-up, and there is no sign of recurrence.

## Discussion

Despite the available literature that AOT is a mixed odontogenic tumor, it also shows the existence of a small number of cells that secrete collagen types I and III and express mesenchymal proteins [9]. The majority of patients with AOT are young, particularly in the second decade, and they tend to be female [10]. The site that is most common is the anterior maxillary area. AOT can be central (intraosseous) or peripheral (in the soft tissue). The intraosseous type is radiographically again classified as follicular (73%), which is linked with an impacted tooth, and the extrafollicular type (24%), which is not associated with the tooth. The peripheral type is quite rare with a prevalence of just 3% [11]. Among all the types, the follicular variety is the most common one; nevertheless, extrafollicular and peripheral cases are also reported in the literature [12-14].

Regarding the nature of AOT, there is controversy about whether it is a hamartoma or a genuine neoplasm, and odontogenic origin is seldom disputed [13]. Its odontogenic origin is supported by its occurrence in the tooth-bearing region and cytological characteristics. While the lesion's restricted size and lack of recurrence in the majority of cases support hamartoma, the lesion's notable size in some of the documented cases supports it as a benign neoplasm not malignant in origin. The studies regarding the genetic profile of AOT mentioned that AOT is associated with RAS mutations and has indolent behavior [15].

With reference to clinical features, the most common location for AOT is the maxillary anterior region especially associated with the maxillary canine. The current case adhered to the widespread intraosseous placement in the maxillary anterior region, which is a biological trend. Given the similarities in clinical symptoms between AOT and dentigerous cysts, AOT is referred to as the perfect imitator [16].

There is a term "cystic AOT" that may arise from dentigerous cysts, and contrary to classic AOT, it is common in males, behaves aggressively, and is more prone to recurrences [17]. Chaves et al. reported multiple AOTs in a patient with Schimmelpenning syndrome [18].

Radiographically, AOTs are usually unilocular well-defined radiolucent lesions with or without sclerotic borders. There is the presence of calcified foci in the radiolucency that imparts snowflakes' appearance. The follicular variant presents as a pericoronal radiolucency and should be differentiated from other pericoronal conditions such as dentigerous cysts, odontogenic keratocysts, calcifying epithelial odontogenic cysts, and tumors.

Although the maxillary canine is the second common site for dentigerous cysts, the peculiar feature of dentigerous cysts is its attachment to the cementoenamel junction of the unerupted tooth, thus presenting as pericoronal radiolucency. Another point is that, though AOT is rarely associated with maxillary lateral incisors, there is evidence of such cases with the follicular variety of AOT associated with maxillary lateral incisors in the available literature. Nevertheless, the case reports describing follicular AOT with mandibular lateral incisors are also documented [19,20]. Pawar et al., in 2022, presented a case of AOT associated with an impacted maxillary lateral incisor in a 12-year-old female that mimicked a dentigerous cyst same as that of the present case. The clinical and radiographic findings of this case were imitating a dentigerous cyst or unicystic ameloblastoma, but the histopathological confirmation was AOT [19]. In AOT, radiolucency may extend apical to the cementoenamel junction. The justification for this is the difference in their pathogenesis. The development of dentigerous cysts is due to the collection of fluid between the crown and reduced enamel epithelium, while AOT develops from odontogenic remnants. Odontogenic keratocyst grows along the medullary space without causing buccolingual expansion. Nevertheless, AOT being a benign tumor may cause buccal/labial and palatal/lingual expansion. In two-thirds of the AOT patients, discrete or flocculent radiopaque foci are also seen, and IOPA is better than orthopantomogram (OPG) at displaying these alterations [21].

It is necessary to confirm the diagnosis of AOT by histopathological examination. There are reports depicting histopathological diversity in the literature. In the literature, up to 20 distinct AOT histological patterns have been reported. It is possible that all of these patterns are merely parodies of the enamel organ [16]. Irrespective of all these patterns, it is interesting that the biological behavior of the tumor remains unchanged. Also, histology has consistently stayed the same throughout history. This unique histomorphology makes it easy to diagnose patients in every situation [16].

## Conclusions

An uncommon benign odontogenic tumor called follicular AOT should be taken into account when making a differential diagnosis of unilocular pericoronal radiolucency in the maxillary anterior area in young females. Management is surgical with a conservative approach. The prognosis is good, and usually, there is no recurrence.
